# Supplementary material for: Vestibular Nuclei: A New Neural Stem Cell Niche?
Source: Cells. 2022 Nov 14;11(22):3598. doi: 10.3390/cells11223598 (PMC9688605; doi:10.3390/cells11223598)
Supplement: Supplementary file 1 [file cells-11-03598-s001.zip › cells-1971116-supplementary.pdf]

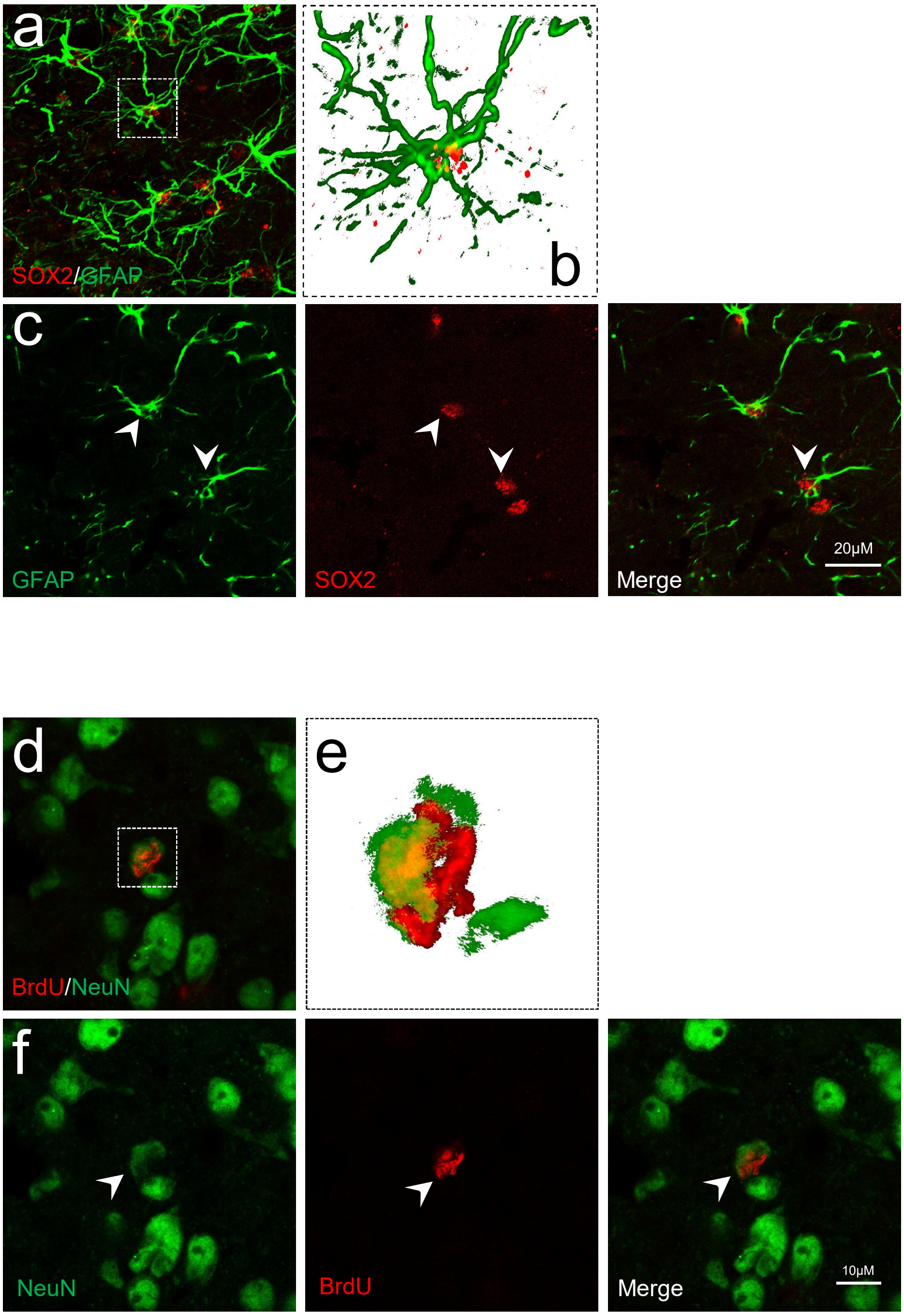

Supplementary Figure S1: Z-stack and 3D reconstruction of SOX2/GFAP and BrdU/NeuN labelling
(a): Orthogonal projection of confocal Z-stacks (21.343µM, 57 slices) stained with SOX2 and GFAP in the medial vestibular nucleus. **(b)**: 3D reconstruction of the cell presented in the dotted square. **(c)**: Single Z-slices (36/57) show colocalization of SOX2 (red) and GFAP (green). Arrows indicate neural stem cells with collocalization of SOX2 and GFAP staining. Scale bar= 20µM. **(d)**: Orthogonal projection of confocal Z-stacks (8.183µM, 18 slices) stained with BrdU and NeuN in the medial vestibular nucleus. **(e): (b)**: 3D reconstruction of the cell presented in the dotted square. **(f)**: Single Z-slices (11/18) show colocalization of BrdU (red) and NeuN (green). Arrows indicate newly generated neuron with collocalization of BrdU and NeuN staining. Scale bar = 10µM.
